# Texture Analysis of Enhanced MRI and Pathological Slides Predicts EGFR Mutation Status in Breast Cancer

**DOI:** 10.1155/2022/1376659

**Published:** 2022-05-26

**Authors:** Tianming Du, Haidong Zhao

**Affiliations:** Department of Breast Surgery, Second Affiliated Hospital of Dalian Medical University, 467, Zhongshan Road, Shahekou District, Dalian, Liaoning 116023, China

## Abstract

**Objective:**

Image texture information was extracted from enhanced magnetic resonance imaging (MRI) and pathological hematoxylin and eosin- (HE-) stained images of female breast cancer patients. We established models individually, and then, we combine the two kinds of data to establish model. Through this method, we verified whether sufficient information could be obtained from enhanced MRI and pathological slides to assist in the determination of epidermal growth factor receptor (EGFR) mutation status in patients.

**Methods:**

We obtained enhanced MRI data from patients with breast cancer before treatment and selected diffusion-weighted imaging (DWI), T1 fast-spin echo (T1 FSE), and T2 fast-spin echo (T2 FSE) as the data sources for extracting texture information. Imaging physicians manually outlined the 3D regions of interest (ROIs) and extracted texture features according to the gray level cooccurrence matrix (GLCM) of the images. For the HE staining images of the patients, we adopted a specific normalization algorithm to simulate the images dyed with only hematoxylin or eosin and extracted textures. We extracted texture features to predict the expression of EGFR. After evaluating the predictive power of each model, the models from the two data sources were combined for remodeling.

**Results:**

For enhanced MRI data, the modeling of texture information of T1 FSE had a good predictive effect for EGFR mutation status. For pathological images, eosin-stained images can achieve a better prediction effect. We selected these two classifiers as the weak classifiers of the final model and obtained good results (training group: AUC, 0.983; 95% CI, 0.95-1.00; accuracy, 0.962; specificity, 0.936; and sensitivity, 0.979; test group: AUC, 0.983; 95% CI, 0.94-1.00; accuracy, 0.943; specificity, 1.00; and sensitivity, 0.905).

**Conclusion:**

The EGFR mutation status of patients with breast cancer can be well predicted based on enhanced MRI data and pathological data. This helps hospitals that do not test the EGFR mutation status of patients with breast cancer. The technology gives clinicians more information about breast cancer, which helps them make accurate diagnoses and select suitable treatments.

## 1. Introduction

Breast cancer is the most common cancer in women in both developed and developing countries. In 2020, there were 2.26 million new cases of breast cancer worldwide, accounting for 11.7% of all cases, and 685,000 deaths, accounting for 6.9% of all cases [1]. Many endogenous and exogenous factors have been identified as being associated with breast cancer etiology. Age is the strongest risk factor for the disease. More than two-thirds of new cases occur after the age of 55, with a higher risk of 4.0 for women older than 65 compared to those younger than 65. Several other risk factors have also been identified in relation to breast cancer. Some risk factors are constant, such as age and mutations in the BRCA1 and BRCA2 genes, which are estimated to account for 20% to 40% of inherited breast cancers with harmful mutations in BRCA1 and BRCA2 [2]. Family and reproductive history are also important risk factors. Other factors are dynamic, such as high endogenous estrogen, hormone therapy, obesity, and alcohol consumption [3, 4]. Among all kinds of diagnostic techniques, imaging techniques are the main diagnostic method used and can provide valuable data for breast cancer patients. It has been shown that various imaging techniques can be used to diagnose and monitor patients with different stages of breast cancer [5]. In addition, there are a number of biochemical biomarkers available as new diagnostic and therapeutic tools for breast cancer patients [6]. Among them, epidermal growth factor receptor (EGFR) can be used as a new biomarker for monitoring the diagnosis and treatment of breast cancer patients [7].

EGFR is one of the four members of the HER family receptors, which are composed of EGFR, HER2, HER3, and HER4. EGFR signaling cascades are key regulators of cell proliferation, differentiation, division, survival, and cancer development. It is expressed in a variety of cancers, including breast, brain, lung, and prostate cancer. In breast cancer, EGFR is overexpressed in approximately half of triple-negative breast cancer (TNBC) and inflammatory breast cancer (IBC) patients [8]. EGFR is one of the first important targets identified for novel antitumor agents. High EGFR expression was identified as an independent predictor of poor outcome in TNBC [9]. This may be because the abnormal expression of the EGFR kinase domain may have an important impact on therapeutic resistance. Recent studies have shown that targeting EGFR enhances the chemical sensitivity of TNBC cells by reconnecting apoptotic signaling networks in TNBC. Some drugs that target EGFR also enhance the effectiveness of other therapies [10]. A study suggests that anti-EGFR-directed radioimmunotherapy combined with radiosensitizing chemotherapy and PARP inhibitor is more effective in treating triple-negative breast cancer [11]. These studies suggest that EGFR-targeted therapy may play a positive role in TNBC and IBC.

Hematoxylin and eosin (HE) staining has stood the test of time as a standard stain for the histological examination of human tissues [12]. In most hospitals, this technology is used as the primary source of pathological diagnosis. This simple dye combination can highlight the fine structure of cells and tissues. Most organelles and the extracellular matrix are eosinophilic, while the nucleus, rough endoplasmic reticulum, and ribosomes are basophilic. Much research has been done on this technology. Many different studies have improved the technique for different diseases. Different from traditional manual recognition, with the development of digital image processing technology, more research has focused on the digitalization and information extraction of pathological images. A study showed that the normalization of HE staining histological images by cycle-consistent generative adversarial networks could effectively enhance the training effect of the network [13]. Some experiments have also shown that HE staining images can be used to determine the Ki-67 score of breast cancer [14]. In this study, a normalization method was adopted to separate the staining effects of the two dyes, which allowed us to focus more on the medical information reflected by a particular dye [15].

Immunohistochemistry (IHC) is an inexpensive and effective technique that is easily available to most pathologists. It has been considerably used in several tumors. Immunohistochemical detection of EGFR is a well-established technique in non-small-cell lung cancer. IHC has good specificity and fairly good sensitivity to mutation-specific antibodies for common EGFR mutations. Moreover, IHC tests can accurately predict responses to EGFR-tyrosine kinase inhibitors (TKIs) [16]. This has been proven in both biopsies and cell blocks [17]. In breast cancer, this method is also widely used. Advances in clinical IHC have greatly improved the ability of clinicians to access information in terms of cost and efficiency. In oncology, these new techniques are highly reproducible, providing a useful alternative to and adjunct to molecular detection. IHC has become a valuable tool in modern cancer treatment.

Enhanced magnetic resonance imaging (MRI) is a highly sensitive breast imaging detection method. Compared with molybdenum target and ultrasound, enhanced MRI has higher resolution and can observe the tissue perfusion status. Different functional MRI sequences can be used to measure spatial differences in cell density, tissue structure, perfusion, and metabolism. With the improvement of medical treatment, enhanced MRI has become widely used as a routine examination for patients highly suspected of having breast cancer. Studies on MRI have shown that the texture features of MRI images can reflect a series of clinical information, such as molecular typing, prognosis, and gene mutations of the tumor itself. MRI-based radiomics analysis has been used to predict mutations in thoracic spinal metastases tumors in lung adenocarcinoma patients [18]. In gliomas, MRI imaging data and radiometric analysis based on these data were also used to analyze the EGFR mutation status of tumors [19, 20]. Another study showed that enhanced MRI data provided a better predictive model for head and neck squamous cell tumors [21]. The above research indicates that it is feasible to construct a prediction model for the texture analysis of image data by using enhanced MRI as the data source. In this experiment, diffusion-weighted imaging (DWI), T1 fast-spin echo (T1 FSE), and T2 fast-spin echo (T2 FSE) were selected to explore their prediction ability for EGFR status. DWI is the only noninvasive method to detect the diffusion of water molecules in living tissues. T1 and T2 sequences ensure that the image data can cover most of the tumor information.

Texture analysis can quantify complex medical image information. With the development of technology, many computer-aided diagnosis methods represented by texture analysis, radiomics, and deep learning play an important role in medical research [22–24]. Specifically, regional heterogeneity can be represented by texture features calculated using a variety of mathematical methods to assess the gray intensity of pixels, with 2D or higher texture features providing more complex information for tumor characterization than simple first-order histogram analysis. Such differences in features can quantify the heterogeneity of the tumor itself and can be used to predict the clinical information of the tumor. The texture analysis of the tumor can classify the tumor itself [25]. Compared with the widely used neural network in medical image field, texture analysis can directly reflect the correlation between parameters and labels. But this also means that this method is not suitable for the classification of complex parameters [26]. The technique could also be used to predict certain genetic mutations in tumors and patient outcomes [27, 28]. This means that texture analysis can provide supplementary information and guidance throughout patient care. Among many texture information extraction methods, using gray level cooccurrence matrix is the most common method. This method has been applied in many tumor images [29]. In this study, we used texture analysis to model information images from multiple sources individually or jointly and explored methods that could predict tumor EGFR mutation status by comparing the models.

## 2. Materials and Methods

### 2.1. Study Participants

The radiology database of the Second Affiliated Hospital of Dalian Medical University was reviewed. We identified 187 patients who underwent MRI from March 2018 to June 2021. Fifty-one patients were excluded owing to incomplete image sequences. Moreover, 22 patients were excluded because of the absence of distinct EGFR results. Therefore, a total of 114 patients were included in this study. The inclusion criteria were as follows: (i) no breast diseases before imaging examination and (ii) did not receive treatment for breast cancer or may have artificially changed breast imaging. The patients underwent MRI examinations before surgery and were diagnosed as grades 3, 4, or 5 according to the Breast Imaging Reporting and Data System. Surgery or biopsy was performed within 1 week to confirm the primary breast cancer diagnosis and EGFR status. We excluded specific breast malignancies, such as IBC, Paget's disease, and breast cancer due to metastasis. Moreover, men and pregnant women were excluded. There was no specific information about the patients in the study, so the study did not involve ethical issues.

### 2.2. Enhanced MRI

We used the American GE 1.5 T Signa HDxt MRI scanner, and the receiving coil was a special one for the surface of the breast. In the prone position, the bilateral breasts naturally hung in the concave hole of the coil. The scan sequence and parameters were as follows: DWI, *b* − value = 800 s/mm, repetition time (TR) = 5,600 ms, echo time (TE) = 74.4 ms, matrix = 130 × 128, field of view (FOV) = 33 cm × 33 cm, and layer thickness = 5 mm. All patients underwent dynamic contrast-enhanced MRI following DWI sequence scanning. Gadolinium diamine was used as the contrast agent. The injection volume was 0.2 mmol/kg, and the flow rate was 2 ml/s to 3 ml/s. Following injection, 20 ml of normal saline was used to flush the tube. We performed continuous nonstop scanning. T1-weighted image plain scanning was initially performed. Following gadolinium injection, we continuously scanned nine phases, with 47 s for each phase. A total of 10 phases were scanned. The scanning time was 7 minutes 6 s, and the turning angle was 15°. Other scanning parameters were as follows: TR = 5.1 ms, TE = 2.5 ms to 12 ms, matrix = 320 × 384, FOV = 30.2 cm × 30.2 cm, and layer thickness = 5 mm. Two radiologists with >10 years of experience in breast imaging diagnosis independently interpreted the MRI results. After discussing the images, they reached a diagnostic consensus. All data were transferred to a GE workstation (Advantage Windows 4.5, General Electric, Madison, WI, USA) [30].

### 2.3. HE Staining

Pathological specimens were obtained from the enrolled patients. No data were excluded owing to missing values or ambiguity. Each sample was fixed in a 10% buffered formalin solution. The fixed tissue was dehydrated and cleared in an automatic tissue processor and stained with HE. The samples were first dewaxed in xylene and alcohol. The samples were then stained. Hematoxylin was dyed for 5 minutes, and eosin was dyed for 3 minutes. Finally, the samples were soaked in alcohol and xylene for dehydration and transparency. The slides are fixed with synthetic resin.

### 2.4. IHC

All specimens were fixed with 4% neutral formaldehyde, embedded in paraffin, and continuously sectioned at a thickness of 4 *μ*m. Following IHC staining, the specimens were observed and photographed under a microscope. We used the immunohistochemical SP method to detect the expression of susceptible genes. Specific steps were performed according to the standard instructions, and professional pathologists interpreted the films. We determined the comprehensive staining intensity and the percentage of positive cells. The final results were divided into the following categories: <5% visible staining: -; 5% to 25% visible staining: +; 26% to 50% visible staining: ++; and >50% visible staining: +++ (where - was defined negative and +, ++, and +++ were defined positive).

### 2.5. Texture Analysis

Texture analysis in this study was performed on two medical datasets. Therefore, the image modeling of a single information source not only explores whether it can reflect the expression of EGFR mutation status but also forms a weak classifier for the final modeling.

#### 2.5.1. MRI Texture Analysis

The Pydicom library of Python was used to read the spatial location of the image. First, we used nearest neighbor interpolation to process the images so that they had equal pixel spacing, followed by image registration according to the spatial location. The region of interest (ROI) was manually obtained on a high-signal DWI sequence, and there was no necrosis or cystic components under ideal circumstances. In the case of no satisfactory image (usually because of low resolution), the ROIs were drawn by referencing T1 images. After standardizing the images, the Laplacian of Gaussian filter was used to process images. We used software to extract texture information, including Elongation, Flatness, LeastAxisLength, MajorAxisLength, Maximum2DDiameterColumn, Maximum2DDiameterRow, Maximum2DDiameterSlice, Maximum3DDiameter, MeshVolume, MinorAxisLength, Sphericity, SurfaceArea, SurfaceVolumeRatio, and VoxelVolume. The extraction of these parameters was based on gray level cooccurrence matrix (GLCM).

#### 2.5.2. Pathology Image Texture Analysis

The scikit-image library of Python was used to process the pathology images. An image normalization algorithm was used to separate the dyeing effects of the two dyes and simulate two dyeing images. This method detects a color vector that meets a specific condition and converts it to a fixed value. On this basis, we extract several features of the image, including contrast, dissimilarity, homogeneity, energy, correlation, and ASM. The extraction of these parameters was also based on GLCM.

### 2.6. Modeling Method

For enhanced MRI images, we selected DWI, T1 FSE, and T2 FSE to extract texture features from the ROI. For pathological images, we extracted texture features from two simulated staining images. For the texture features obtained from each image, we selected features with a strong correlation with EGFR expression and adopted methods including adaboost, support vector machine (SVM), random forest, and decision tree for modeling. We selected the model with the best effect and compared it with the model from the same data source (MRI/HE). We selected the model with the best effect (with higher AUC value) as the weak classifier. In the case of similar classification effects, we chose the overfitting model as the weak classifier. The choice was made based on the advice of a software architect. Finally, we integrated the weak classifiers of the two data sources to judge EGFR mutation status.

## 3. Result

### 3.1. Clinical Features

The clinical information of the patients is shown in Table 1.

### 3.2. Enhanced MRI Texture Analysis

#### 3.2.1. DWI

**Figure 1:**
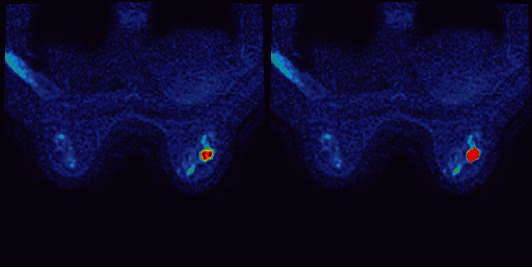
Example of DWI.

**Figure 2:**
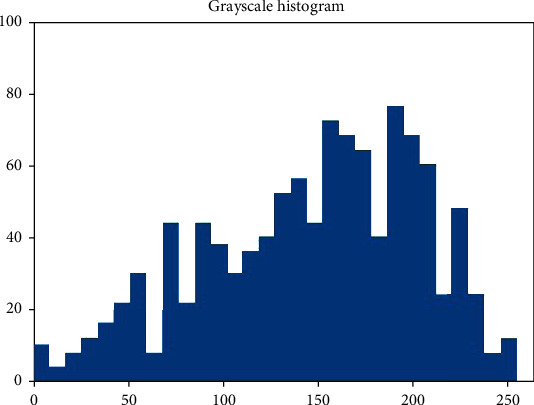
Grayscale histogram of pixels in the ROI of DWI.

According to the ROI of the image (Figure 1), the gray level of the image was measured. According to this, a grayscale histogram was made (Figure 2).

A pseudocolor map of a 37-year-old patient with EGFR-positive mutation status (T1N1M0, ER: +, PR: +, HER-2: +, Ki67: -) is shown. Following imaging diagnosis, the breast tumor (considered malignancy) was confirmed by postoperative pathology. The images of T1 FSE and T2 FSE sequences and HE staining images mentioned below are also from this patient. The red part of the right picture is the ROI.

**Figure 3:**
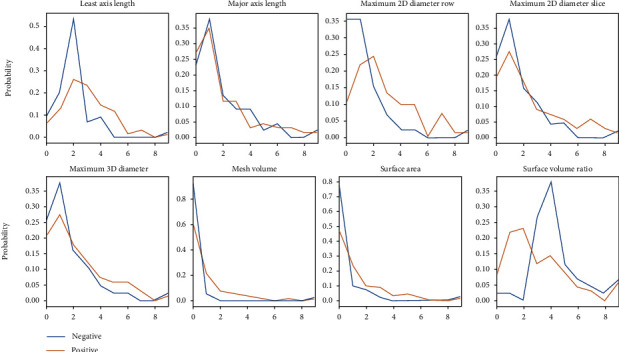
Feature distribution in DWI.

In the DWI sequence, we selected some features for modeling. As shown in Figure 3, these parameters had significant distribution differences according to the mutation status of EGFR in patients.

After comparing the modeling effects of various methods, we found that the adaboost algorithm had a relatively good classification effect on this problem (test group: AUC, 0.735; specificity, 0.9286; and sensitivity, 0.5238).

#### 3.2.2. T1 FSE

**Figure 4:**
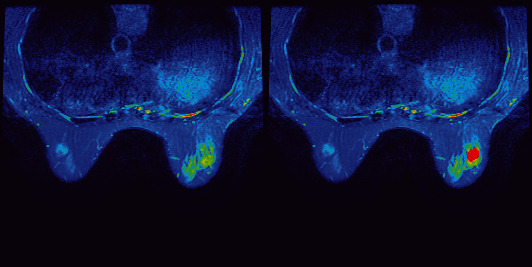
Pseudocolor maps of T1 FSE.

**Figure 5:**
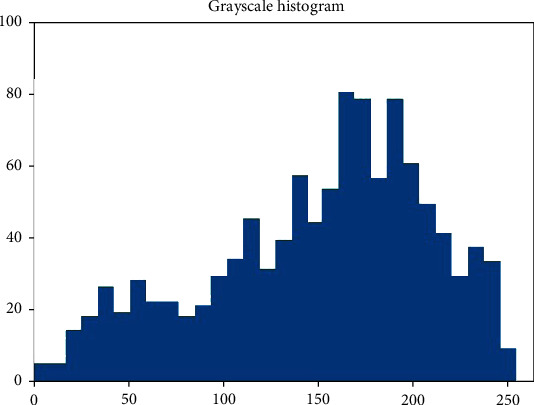
Grayscale histogram of pixels in the ROI of T1 FSE.

According to the ROI of the image (Figure 4), the gray level of the image was measured. According to this, a grayscale histogram was made (Figure 5).

**Figure 6:**
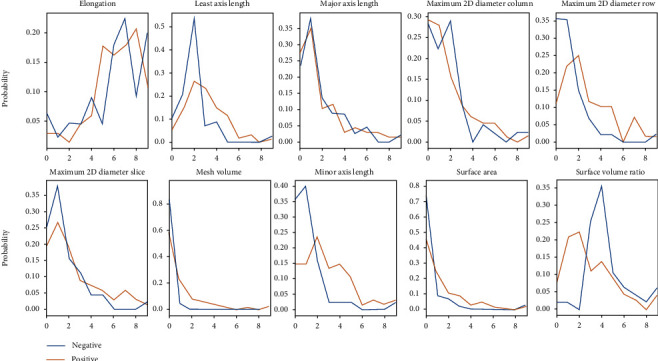
Feature distribution in T1 FSE.

In the T1 FSE sequence, we selected some features for modeling. As shown in Figure 6, these parameters had significant distribution differences according to the mutation status of EGFR in patients.

After comparing the modeling effects of various methods, we found that the adaboost algorithm had a relatively good classification effect on this problem (test group: AUC,0.741; specificity, 0.8571; and sensitivity, 0.6667).

#### 3.2.3. T2 FSE

**Figure 7:**
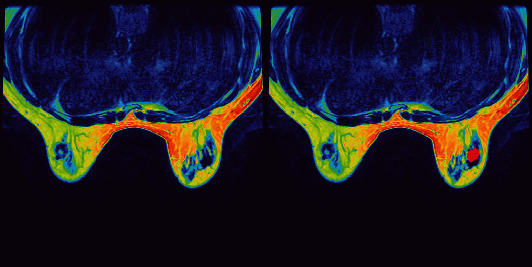
Pseudocolor maps of T2 FSE.

**Figure 8:**
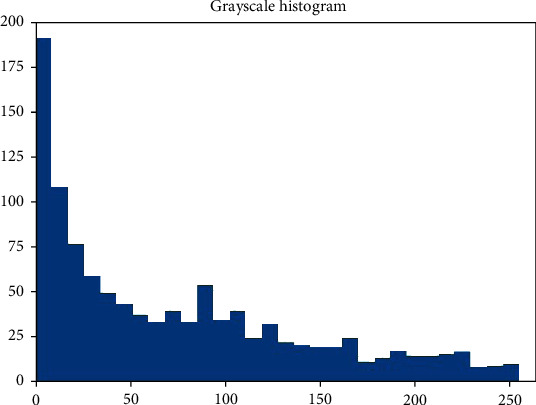
Grayscale histogram of pixels in the ROI of T2 FSE.

According to the ROI of the image (Figure 7), the gray level of the image was measured. Based on this, a grayscale histogram was made (Figure 8).

**Figure 9:**
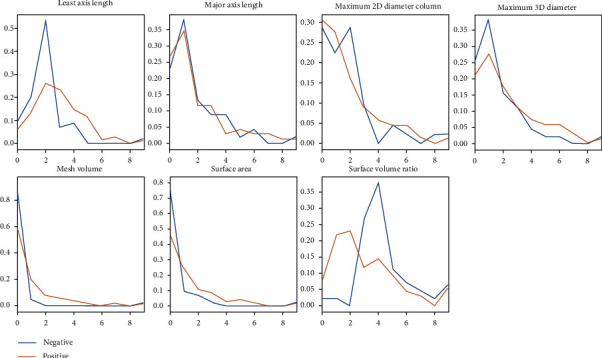
Feature distribution in T2 FSE.

In the T2 FSE sequence, we selected some features for modeling. As shown in the figure (Figure 9), these parameters have significant distribution differences according to the mutation status of EGFR in patients.

After comparing the modeling effects of various methods, we found that the decision tree algorithm had a relatively good classification effect on this problem (test group: AUC,0.726; specificity, 0.6429; and sensitivity, 0.8095).

**Figure 10:**
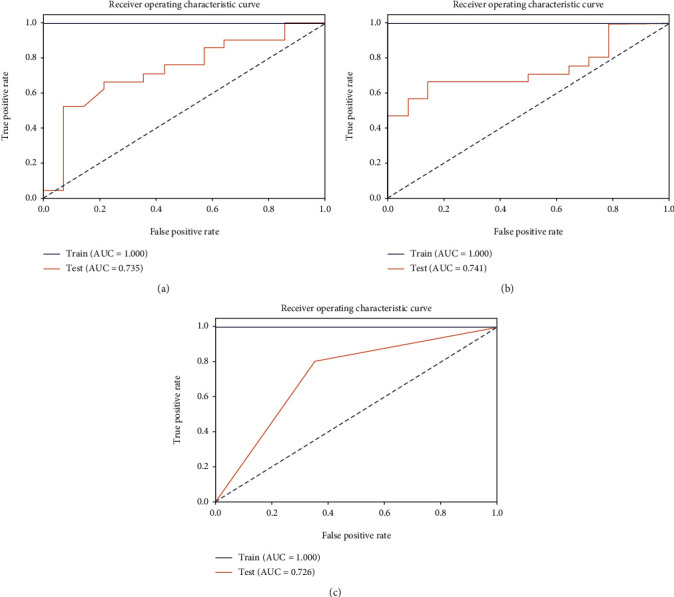
ROC curves of the three classification models ((a) DWI, (b)T1 FSE, and (c) T2 FSE).

By comparing the three models based on enhanced MRI, we conclude that the model based on T1 FSE is more suitable for weak classifiers (Figure 10).

### 3.3. Pathological Image

**Figure 11:**
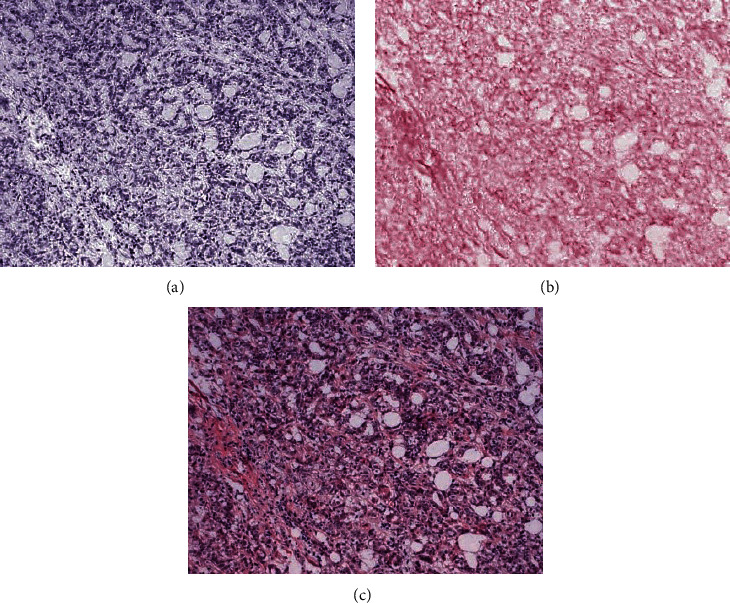
Simulated staining images ((a) hematoxylin, (b) eosin, and (c) HE).

We used a special normalization algorithm to normalize the pathological images of patients. This method can separate the staining effect of two dyes (Figure 11).

### 3.4. Hematoxylin

In the simulated hematoxylin staining image, we selected contrast, dissimilarity, homogeneity, energy, correlation, and ASM as the features. Finally, we chose the decision tree algorithm for modeling, which had a relatively good classification effect on this problem (test group:AUC,0.595; specificity, 0.4286; and sensitivity, 0.7619).

### 3.5. Eosin

In the simulated eosin staining images, we also selected contrast, dissimilarity, homogeneity, energy, correlation, and ASM as the features. We finally chose the random forest algorithm for modeling, which had a relatively good classification effect on this problem (test group; AUC,0.662; specificity, 0.7857; and sensitivity, 0.6667).

**Figure 12:**
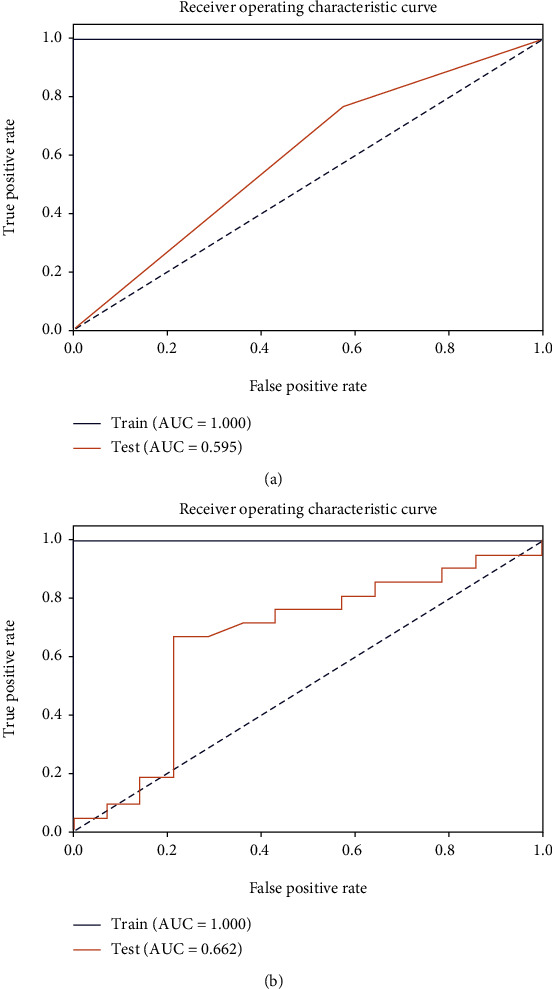
ROC curves of the two classification models ((a) hematoxylin and (b) eosin).

By comparing two models based on pathological images, we conclude that the model based on eosin images is more suitable for weak classifiers (Figure 12).

**Figure 13:**
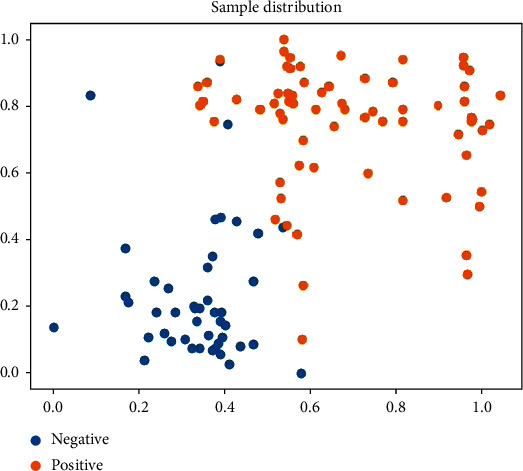
Scatter diagram of data distribution.

Finally, we selected two models from the MRI and pathological models (T1 FSE model and eosin model). As seen from the data distribution of the predicted values of the weak classifiers, EGFR mutation status can be clearly distinguished (Figure 13).

**Figure 14:**
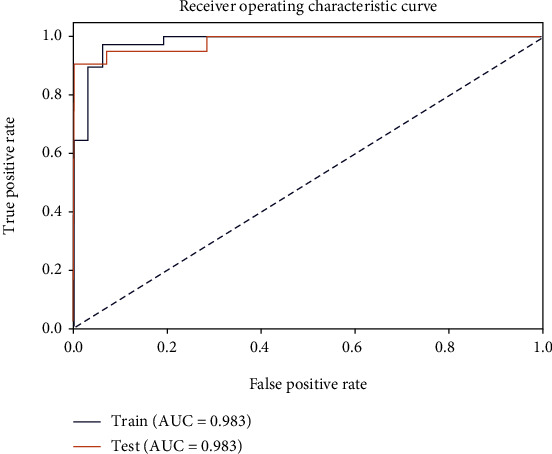
ROC curves of the final classification models.

Finally, we adopted SVM to classify the data. In both the training set and the test set, the model achieved good results (Figure 14) (training group: AUC, 0.983; 95% CI, 0.95-1.00; accuracy, 0.962; specificity, 0.936; and sensitivity, 0.979; test group: AUC, 0.983; 95% CI, 0.94-1.00; accuracy, 0.943; specificity, 1.00; and sensitivity, 0.905).

## 4. Discussion

In this study, we developed an algorithm to predict EGFR expression in patients with untreated breast cancer based on enhanced MRI and pathologic images. This provides additional clinical information for physicians who do not perform EGFR testing. Given the growing importance of EGFR in the diagnosis and treatment of breast cancer, this approach is certainly valuable.

The EGFR gene is an oncogene-driven gene [31]. EGFR has several carcinogenic effects, including the stimulation of DNA synthesis, cell cycle, cell proliferation, cell metastasis, and invasion. Moreover, EGFR mutation was discovered to be the first molecular change in lung cancer. TKIs that target sensitizing mutations in the EGFR gene are a key pillar of the treatment of non-small-cell lung cancer [32]. To date, acquired resistance to EGFR-TKIs has been an inevitable process, usually occurring 9-12 months after treatment [33]. In breast cancer, there are also some studies describing the importance of EGFR mutation status in the diagnosis and treatment of breast cancer. Current evidence suggests an association between low baseline serum EGFR and shorter survival or reduced treatment responses in patients with advanced breast cancer. EGFR is usually overexpressed in metastatic breast cancer, but metastatic breast cancer is usually resistant to EGFR therapy. Anti-EGFR therapies such as cetuximab and erlotinib have had limited efficacy in clinical trials [34]. Therefore, more studies are needed to understand the underlying mechanistic link between EGFR expression and metastasis progression.

Recent studies on the texture analysis of EGFR mutation status mainly focused on lung cancer [35]. This is because EGFR-related targeted drugs have played an important role in the diagnosis and treatment of lung cancer [36]. Due to the particularity of lung tumor diagnosis, CT is often the most important imaging method for patients [37]. Therefore, the main image data sources for the texture analysis of EGFR expression are CT or PET/CT [38]. In the field of breast cancer, only a few studies have chosen to use MRI for analysis. Therefore, there is quite a gap in this area. However, in the above studies, due to the difficulty in obtaining medical records, it is rare to comprehensively consider pathological images and imaging data. In addition, many assays have been proposed to detect EGFR. A noninvasive test proved to have great potential [39]. In our experiment, we used medical information images from two different sources to predict EGFR expression. This multidimensional modeling method undoubtedly greatly improves the accuracy of the prediction results.

In this study, we adopted a specific method of pathological image normalization. This method helps us to exclude the influence of different dyeing conditions on the image coloring. At the same time, this normalization method separates the dyeing effect of the two dyes. The two dyes have different affinity for different tissues. This method gives us the ability to analyze the texture characteristics of specific tissues. Prior to this, we had conducted an imaging study on breast cancer [30]. The enhanced MRI data in both studies came from the same machine and included roughly the same patients. We can obviously see that in the aspect of imaging data analysis, compared with the simple texture analysis, the method of tumor image segmentation undoubtedly has higher accuracy.

However, this experiment still has considerable limitations. First, in the process of making pathological sections, tumor tissues will shrink to different degrees after chemical process. This means that the spatial structure of the tumor has changed. Therefore, it is extremely difficult to complete the spatial correspondence between tumor images and image data. It is a very difficult task to try to analyze the tumor pathological sections and corresponding image layers together. This makes it impossible to compare our models across image sources. This difficulty forced us to separate models from different sources of clinical information, which undoubtedly reduced the efficiency of model screening. If images from different sources can correspond spatially, it means that we can give a higher information dimension to the space represented by each voxel. So, on the one hand, we can reduce the number of models. On the other hand, we were able to explore the effect of intratumor heterogeneity on EGFR status. We can even explore MRI's ability to distinguish tissue distribution by using artificial methods to distinguish tumor subareas and boundaries reflected in pathological images. On this basis, it is very potential to explore a high-precision tumor subregion segmentation method [40]. Second, it is well known that tumors are heterogeneous diseases, and the composition of each subregion can well reflect the characteristics of tumors. This imaging method of analyzing the characteristics and relationships between tumor subareas is medically known as habitat imaging. However, due to the chemical process of pathological sections, the proportions of mesenchymal and parenchymal cells in each subregion are significantly different, and the contraction of each subregion is undoubtedly different after such treatment. This means that the spatial structure of the original subregion of the tumor has been artificially altered [41]. This change has a significant impact on habitat imaging that emphasizes relationships between subregions. At the same time, this method of research requires the acquisition of overall tumor image data. For pathological sections, it is undoubtedly very difficult to obtain such a large number of complete sections, including tumor edge conditions. Therefore, in this experiment, we only selected representative tumor sections as information sources for texture analysis. This choice also brings problems. Compared with the image data generated by artificial ROI, the overfitting of the model constructed by pathological images is greater. This means that the biopsy does not cover all the information about the tumor. With the development of computer-aided diagnosis (CAD) and image scanning technology, whole-slide image (WSI) scanners are widely used in the field of pathological diagnosis. Therefore, WSI analysis has become the key to modern digital histopathology. In this study, we used pathological images artificially generated by pathologists to ensure that the selected areas were dominated by tumor components. Due to objective conditions, this study did not use WSI as the information source for pathological images [42]. Modeling based on pathological staining images is significantly worse than modeling based on enhanced MRI data. The source of such pathological images may not adequately reflect the tumor itself. The total number of patients included in this experiment was limited due to the hospital scale and total number of patients. However, the final modeling results showed a better performance. Nevertheless, the strong overfitting characteristic of a weak classifier is undoubtedly the embodiment of insufficient data [43]. In addition, the feature extraction method adopted in this paper is relatively simple [44].

The imaging features used in this study are from GLCM formed by a single image processing method [45]. In fact, the features extracted by the texture analysis technique can be combined with a variety of filters to extract the texture information of a variety of matrices [46]. However, in our study, we found that the effect of a single model can be improved to some extent by simply adding the dimensions of texture parameters. Nevertheless, when we selected such a model as a weak classifier, the effect of the final model did not have more advantages, and its data distribution did not show more obvious differences. This finding is in accordance with the software architect's suggestion that the overfitted weak classifier will not have a significant effect on the final model.

The method adopted in this study is to model the texture features extracted from images. This usually entails converting the original image to a grayscale image for further manipulation. However, some experiments show that RGB feature extraction technology has achieved good results in a variety of malignant tumors, including breast cancer and prostate cancer [47]. In recent years, artificial intelligence (AI) technology has developed rapidly. In particular, important achievements have been made in computer vision, image processing, and analysis. In pathology, there are many studies showing the use of multiple neural networks to classify and segment tumors [48, 49]. A study showed that convolutional neural networks have also made a breakthrough in the recognition of EGFR [50]. In fact, in the field of medicine, convolutional neural network has become a hot topic. From tumor image recognition to gene expression classification, convolutional neural network has made great contributions to medical research [51, 52]. In a preexperiment in this paper, we discussed the predictive power of using deep learning for EGFR mutation status. There is no doubt that deep learning can make more effective use of information in images. At present, the data sources of relevant studies mainly focus on using pathological images or radiological images alone. One feasible idea is to use Markov random field or conditional random field model to classify medical images directly [53]. Both methods have their own advantages in information processing [54, 55]. This method has made progress not only in the identification of EGFR status or breast cancer but also in gastrointestinal tumors, gliomas, reproductive system tumors, respiratory system tumors, etc. [56–58]. In both qualitative and quantitative medical problems, this approach has undisputed obvious advantages [59, 60]. Another idea is to use the image segmentation method to subsegment the tumor. These methods include *k*-means, *U*-Net, and other unsupervised or supervised algorithms [61, 62]. The final prediction model is established by describing the formed subregion. This method has made good progress in the field of imaging. All in all, we will try to develop a new comprehensive classification method in the future work. This method can make full use of WSI images and combine with imaging data to classify breast cancer. Neural networks will undoubtedly replace our current texture extraction methods.

## 5. Conclusions

In summary, a model was established based on features extracted from MRI and pathology images. The model can satisfactorily predict EGFR detected by immunohistochemistry. This study provides a reference for screening the high-risk population for surgery, drug therapy, and prognosis.

## Figures and Tables

**Table 1:** Clinical characteristics of patients with breast cancer.

Characteristics	Training group (*n* = 79)	Test group (*n* = 35)
Age (years)	55.3	55.9
ER		
Positive	64	26
Negative	15	9
PR		
Positive	53	25
Negative	26	10
HER2		
Positive	24	7
Negative	55	28
Stage I	55	28
Stage II	17	5
Stage III	4	2
Stage IV	3	0
Lymphonodus		
Positive	25	11
Negative	54	24
EGFR		
Positive	48	21
Negative	31	14

ER: estrogen receptor; PR: progesterone receptor; HERK2: human epidermal growth factor receptor 2; EGFR: epidermal growth factor receptor.

## Data Availability

The MRI and clinic information data used to support the findings of this study are available from the corresponding author upon request.
